# The mouse pathology ontology, MPATH; structure and applications

**DOI:** 10.1186/2041-1480-4-18

**Published:** 2013-09-13

**Authors:** Paul N Schofield, John P Sundberg, Beth A Sundberg, Colin McKerlie, Georgios V Gkoutos

**Affiliations:** 1 Department of Physiology, Development and Neuroscience, University of Cambridge, Downing Street, CB2 3EG, Cambridge, UK; 2 The Jackson Laboratory, 600, Main Street, Bar Harbor, ME 04609-1500, USA; 3 Physiology and Experimental Medicine Research Program, The Hospital for Sick Children, M5G 1X8, Toronto, Canada; 4 Department of Computer Science, University of Aberystwyth, Old College, King Street, SY23 2AX Ceredigion, Wales

**Keywords:** Pathology, Ontology, Disease, Mouse, Phenotype

## Abstract

**Background:**

The capture and use of disease-related anatomic pathology data for both model organism phenotyping and human clinical practice requires a relatively simple nomenclature and coding system that can be integrated into data collection platforms (such as computerized medical record-keeping systems) to enable the pathologist to rapidly screen and accurately record observations. The MPATH ontology was originally constructed in 2,000 by a committee of pathologists for the annotation of rodent histopathology images, but is now widely used for coding and analysis of disease and phenotype data for rodents, humans and zebrafish.

**Construction and content:**

MPATH is divided into two main branches describing pathological processes and structures based on traditional histopathological principles. It does not aim to include definitive diagnoses, which would generally be regarded as disease concepts. It contains 888 core pathology terms in an almost exclusively *is*_*a* hierarchy nine layers deep. Currently, 86% of the terms have textual definitions and contain relationships as well as logical axioms to other ontologies such the Gene Ontology.

**Application and utility:**

MPATH was originally devised for the annotation of histopathological images from mice but is now being used much more widely in the recording of diagnostic and phenotypic data from both mice and humans, and in the construction of logical definitions for phenotype and disease ontologies. We discuss the use of MPATH to generate cross-products with qualifiers derived from a subset of the Phenotype and Trait Ontology (PATO) and its application to large-scale high-throughput phenotyping studies. MPATH provides a largely species-agnostic ontology for the descriptions of anatomic pathology, which can be applied to most amniotes and is now finding extensive use in species other than mice. It enables investigators to interrogate large datasets at a variety of depths, use semantic analysis to identify the relations between diseases in different species and integrate pathology data with other data types, such as pharmacogenomics.

## Background

Since the late eighteenth century when achromatic lenses and reliable histological stains began to be available, investigators of anatomic pathology, and particularly in the mid -nineteenth century the innovators of cellular pathology such as Rudolf Virchow, developed and applied terminologies to describe their observations [1,2]. These depended on the “school” to which the pathologists belonged, but more importantly on the etiologic or mechanistic paradigm in which they were working [3]. One of the great achievements of the nineteenth century was the recognition of the universality of pathological processes and entities and their occurrence in multiple species as recognisable manifestations of the same underlying processes [4]. It was, nevertheless, a century before broadly accepted and rationally structured pathology terminologies were developed (e.g. [5]). The development of pathology terminologies has to an extent occurred independently of disease terminologies and nosologies, partly as a result of the much longer history of classifying diseases, and partly due to the inherited preconceptions of the nature of disease in clinical medicine.

The distinction between pathological and clinical descriptions of disease, disorders and predispositions is still not satisfactorily resolved. However, in recent years there have been attempts to rationalise the definitions of these concepts [6] and their relation to each other as part of a broadly applicable model of disease other than an unstructured collection of manifestations or phenotypes which are found in that class of individuals forming the basis of a diagnosis. Issues about severity, time course, organ involvement etc. are beginning to be addressed, but it is remarkable that even treating diseases as a “bag“ of phenotypes has been shown to provide a powerful approach in establishing the relationships between diseases, and the presence of related diseases in different organisms [7-10]. What has recently been identified as important, nevertheless, is that the tissue-specific resolution of the recording of lesions, and the ability to record the pattern of disease within an individual, has proved vital for GWAS mapping of predisposing genetic variants in inbred strains of mice allowing each class of lesion to be analysed in isolation [11,12].

The discipline of pathology may be broken down into clinical and anatomic pathology, the former is concerned with clinical chemistry, hematology, clinical microbiology and emerging sub-specialities such as molecular diagnostics and proteomics. The latter, which forms the domain of MPATH, deals with the histological, histochemical or immunohistochemical observations of alterations in tissue composition or architecture. Both branches of the medical specialty, which are increasingly merging, may be viewed as aspects of phenotyping, and both provide subtypes of the clinical signs associated with ongoing disease processes, the results of developmental abnormalities, or the historical presence of disease.

### Anatomic pathology nomenclature and its applications

The universality of the repertoire of responses to underlying genetic or extrinsic insults means that gross and histopathologically-defined phenotypes are some of the most useful phenotypes for relating diseases between different species, and constitute some of the most species-agnostic phenotype descriptors. This makes a pathologic term-based ontology a crucial tool in experimental and clinical phenotype data capture [13].

The development of systematic human pathologic nomenclatures has been driven by the efforts of the American College of Pathologists, initially with the development of the pathology specific nomenclature (SNOP) over 40 years ago [14] to the current SNOMED –CT with cross references to UMLS, the NCI thesaurus and other terminologies. The ICD [15], now in its 11^th^ revision and the associated ICD-O v-3 for cancer, also contains descriptions of many pathological lesions.

The other driver for pathologic terminology standardisation has been coding of lesions from toxicopathology. The American Society of Toxicopathology (STP) working with Registry of Industrial Toxicology Animal-data (RITA) database group in Europe has produced several internationally accepted nomenclature systems, particularly focusing on proliferative lesions. Recently, the STP has undertaken a major harmonization exercise for rodent pathology – the INHAND (International Harmonization of Nomenclature and Diagnostic Criteria for Lesions in Rats and Mice) initiative [16]. So far this group has reported on the hepaticobiliary, respiratory, nervous and urinary systems [17-20]. For some time the National Cancer Institute’s Mouse Models of Human Cancer consortium (MMHCC) has been examining the classification of tumours in genetically engineered mice. MMHCC has produced a consensus base terminology for neoplasias of the major organ systems that have been presented in a series of papers over the last decade [21].

Despite the huge value of these resources, none is currently constructed as an ontology with meaningful axioms to support inference and automated reasoning, and to that end we developed MPATH to describe lesions that arise in laboratory mice.

### Construction and content

The MPATH ontology was constructed *ab initio* by a group of clinical and veterinary pathologists in 2,000 and has since been revised and augmented by an evolving group of US and European pathologists on a regular basis. It is clear from more than a decade of experience that expert input and manual curation are essential to generate an accurate and functional resource. One strategy for building the ontology has been to integrate it into large-scale phenotyping and diagnostic programs so that the pathologists use it on a daily basis and have fields to add missing terms or synonyms that they are more familiar with thereby constantly increasing its coverage and utilitarian value.

**Figure 1:**
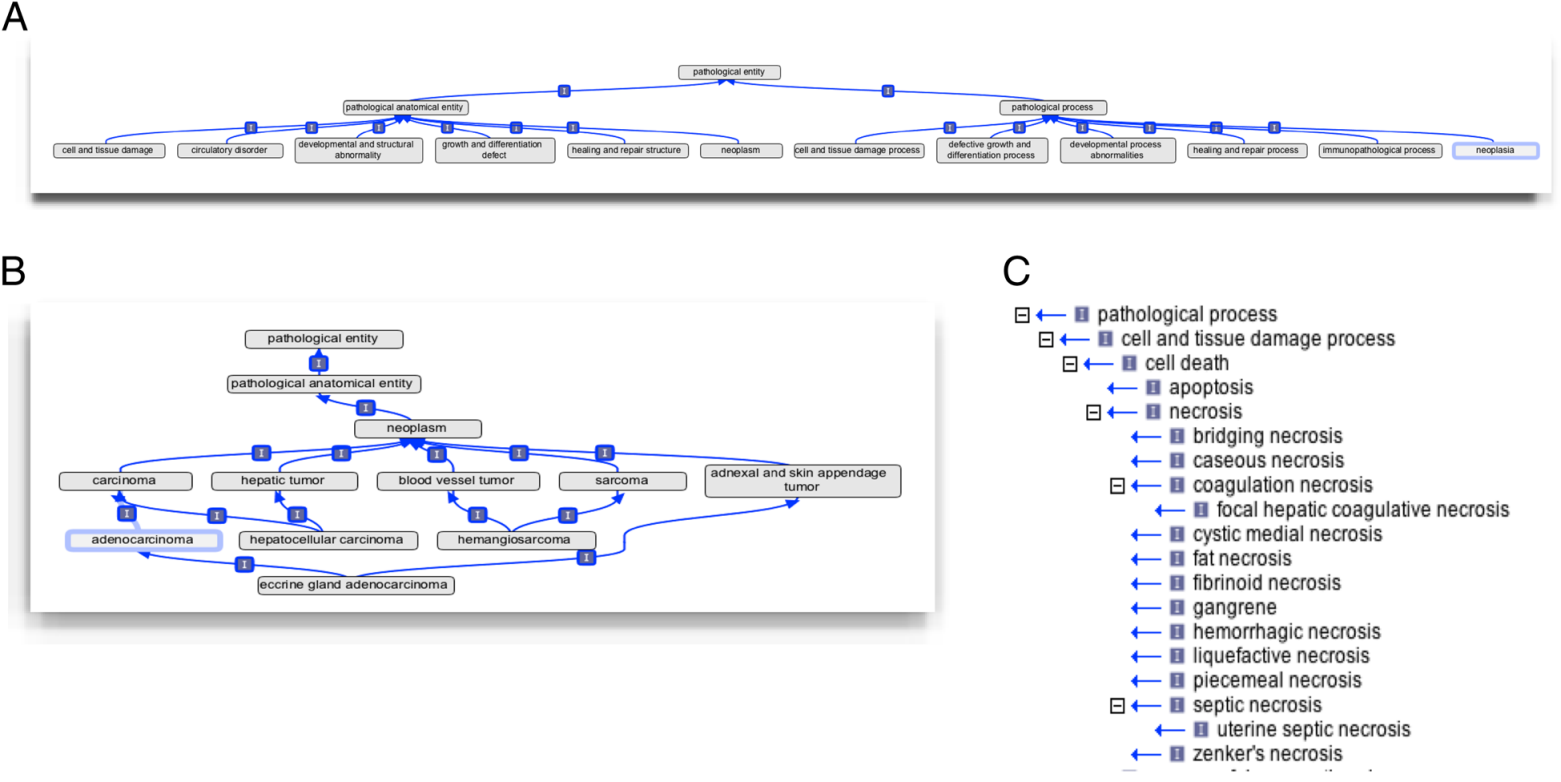
**Structure of MPATH. A**; overall structure of the top level classes in MPATH showing division into pathological process and physical entity for the major divisions. **B**; polyhierarchy showing multiple parentage for some anatomically predicated tumor classes and their anatomical and morphological classification. **C**; cell and tissue damage process segment of MPATH showing main pathological process classes in this branch.

MPATH contains 888 classes in an almost exclusively *is*_*a* hierarchy eight layers deep. *Part*_*of* relations are used only in the case of, for example, adenomatous polyp (MPATH:490) and intraductal papilloma (MPATH:285) where pathologists distinguish between a macroscopic lesion (“–osis”) and individual lesions which make them up; both need to be described. The top level distinction in MPATH is between pathophysiology (pathological processes) and anatomic pathology (pathological entities). One issue met frequently in development was the normal practice of referring to observation of a physical lesion by using the process term; for example “necrosis” or “sclerosis”. Thus the noun describing the real-world entity observed is homonymous with the inferred process. This problem has been addressed through the textual and logical definitions of terms but is a recurrent source of confusion in formal treatments of pathology nomenclature. Pathologists using MPATH almost exclusively use the anatomic pathology segment of the ontology with the exception of describing inflammation or other general processes where the process is described using qualifiers such as “acute” which are logically process-specific, consistent with the use of Phenotype And Trait Ontology (PATO) [22] qualifiers (see below). The upper levels of MPATH’s anatomic pathology branch include six broad domains familiar to all traditions of pathology training and comprehensively covering all known lesions; cell and tissue damage, circulatory disorder, developmental and structural abnormality, growth and differentiation defect, healing and repair structure, and neoplasm, and are as orthogonal as is feasible given the complexities of pathobiology (see Figure 1A). The upper levels of MPATH’s pathophysiology branch denote pathological processes that underlie lesions and include six broad domains; cell and tissue damage process (see Figure 1C), defective growth and differentiation process, developmental process abnormalities, healing and repair process, immunopathological process, and neoplasia. All pathological processes and entities can be placed within these upper level domains, which will be familiar to all pathologists and are common to all amniotes.

#### Relationship to upper level and other ontologies

MPATH is largely congruent with the upper level Ontology for General Medical Science (OGMS) [6], founded on the Basic Formal Ontology (BFO). Pathological bodily process (OGMS:0000061) and pathological anatomical structure (OGMS:0000077) are broadly mappable to the upper levels of MPATH; MPATH:603 (pathological anatomical entity) and MPATH: 596 (pathological process) respectively. However, more detailed mapping is difficult. For example the MPATH experts view congenital malformations as pathological anatomical structures, whereas OGMS views them as distinct, and similarly MPATH views inflammation as a pathological process whereas OGMS does not include this as a pathological bodily process. Until such discrepancies are resolved, integration of MPATH into the OGMS framework will be problematical.

From the point of view of application, the most important mappings for MPATH are to the Human Phenotype Ontology (125), the Mammalian Phenotype ontology (111), the Disease Ontology (231), SNOMED-CT (867) and the NCIt (566), reflecting the emphasis on the domain of anatomic pathology rather than disease.

#### Definitions and axiomatic relationships

Currently, 86% of classes have textual definitions. Each class is in the mouse pathology namespace and is uniquely identified by a URI of the form: http://purl.obolibrary.org/OBO/MPATH_n. The main ontology is available in both the OBO Flatfile Format and the Web Ontology Language (OWL). MPATH is housed in a subversion repository and is made available via OBO registry, Bioportal (http://purl.bioontology.org/ontology/MPATH) and on the project’s website http://mpath.googlecode.com/. MPATH contains relationships and other logical axioms to other ontologies such the Gene Ontology (GO) [23], Cell Type ontology (CL) [24] and the Phenotype And Trait Ontology (PATO) [22]. For example, the MPATH term transitional cell metaplasia (MPATH:172) represents a metaplastic response of the transitional epithelium, for example in the bladder to give squamous metaplasia and glandular metaplasia. To allow computational access to these relations, we use the derives-from relation and relate metaplasia (MPATH:549) (an MPATH term that denotes an abnormal transformation of a differentiated adult cell or tissue of one kind into a differentiated tissue of another kind) with the CL term transitional epithelial cell (CL:0000244).

### Application and utility

#### A post-composition strategy for pathology coding

Traditionally pathologists have relied on a narrative form of recording their definitive diagnoses, making use of morphologic, etiologic, and disease-based terms that collectively provide a diagnosis useful for clinical patient management. This is particularly important for non–neoplastic lesions where it can be complex to capture important subtleties of distribution, severity, microscopic sub-type and anatomical location for example. Whilst this is the gold standard, it is not possible to compute on data recorded in this way and it is very difficult to tabulate and quantitatively analyse the collected information. There are strong arguments, mainly from experience in toxicologic pathology, that a descriptive (anatomic) rather than diagnostic coding is the most objective and useful way to code pathology-based observations. This is particularly relevant to examination of mutant mice where traditional etiologic or summative diagnostic terms are simply not available because of the novelty of the lesion or its presentation. This is particularly the case where mice are manipulated to model human conditions that have not been previously seen, for example lung or mammary tumours [11,25,26] which have not previously been reported to occur spontaneously in mice. In many cases, a disease diagnosis implies a particular pathogenesis or etiology based on the spontaneous disease, which is not appropriate for the disease caused by genetic and sometimes both genetic and external challenge combined. This latter issue is of particular concern to practicing pathologists and in the development of MPATH we have been urged to include some diagnostic terms as well as descriptive anatomic ones.

Many tissue responses are common to multiple anatomical sites and as far as possible the verbosity (ontology “bloat”) of specifying a particular response in multiple tissues has been avoided, with the additional topographical or anatomical information for description coming from an anatomy ontology, generally the MA [27] or EMAP ontologies [28] for the mouse, however, there is often an intrinsic anatomical element embedded in the term or traditional pathology includes information about the cell type or tissue of origin. This is most frequent with the neoplasias and we felt that such terms were best included in their familiar form. Figure 1B shows how anatomically predicated classes such as hepatocellular carcinoma have multiple parents, providing relations in this case to both carcinoma and hepatic tumor superclasses. Most observations made by pathologists using MPATH are, nevertheless, cross-products using a combination of an MPATH term and an anatomical (MA) or cell type (CL) [24,29] component. This strategy provides all of the necessary coverage.

**Figure 2:**
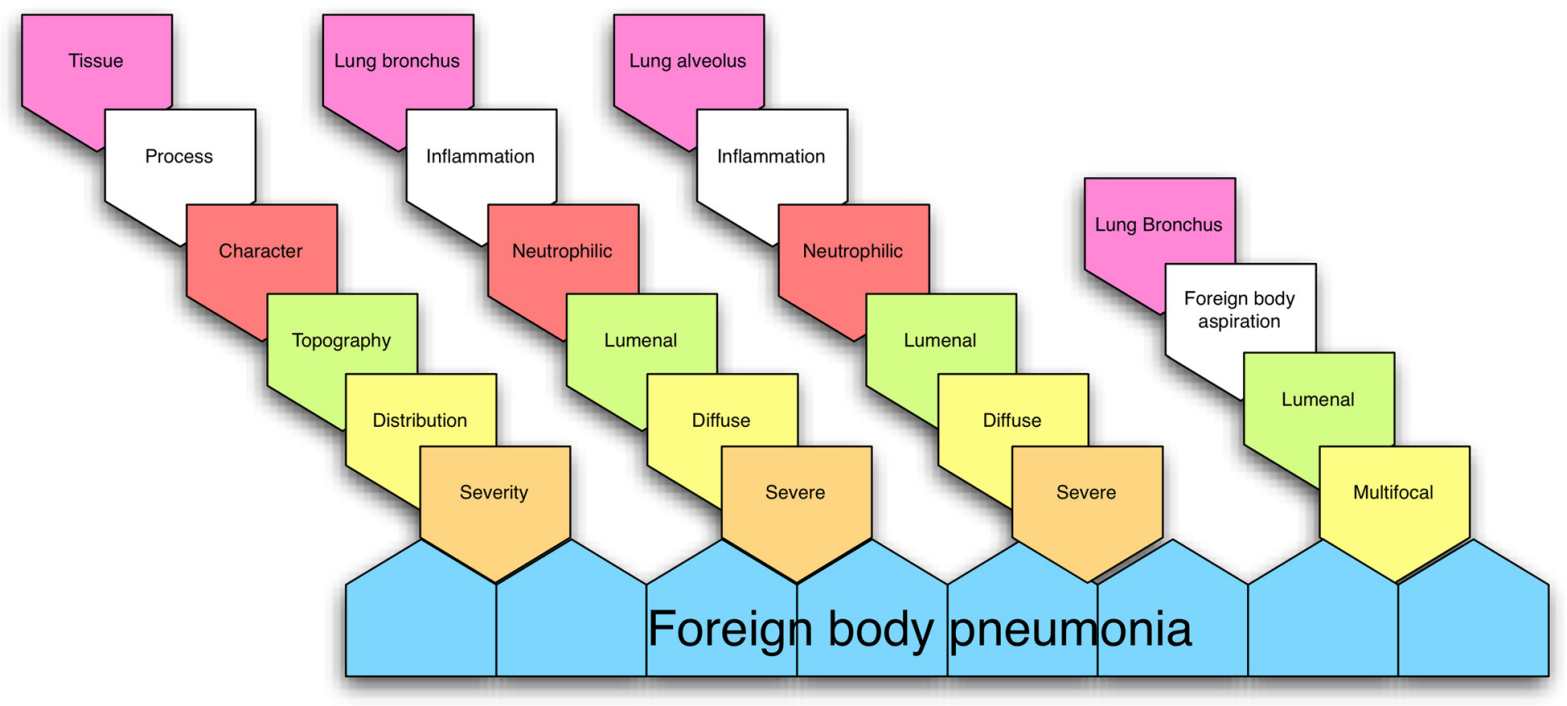
**Post-composition coding strategy.** Elements of the compound description are specified by the string of linked pentagons on the left hand side of the figure and specific examples given for three observations which taken together are indicative of foreign body pneumonia. Tissue terms are taken from the appropriate anatomy ontology, eg. MA, Process from MPATH, character, topology, distribution and severity from PATO. The combination of observations defines the disease foreign body pneumonia.

In addition to the core terms in MPATH, it is important to describe organ-specific topography, distribution, microscopic character, duration/chronicity and severity. These are not included in MPATH but drawn from other ontologies. These qualifiers or modifiers are generally applicable across a wide range of organs and lesions and so need to be coded separately to the core terms to allow post-composition as required. The pattern we have adopted is very close to that recommended by the INHAND proposals and also includes “compound” terms which lie beneath a definitive diagnosis or disease level of description, but bundle defined sets of descriptive terms, for example “nephropathy”, “alopecia”, “glomerulonephritis” which are in common use and well understood. These qualifiers have been incorporated into PATO, and some examples are given in Table 1. The strategy for composing pathology descriptions using the combination of MPATH, MA and PATO is summarised in Figure 2.

**Table 1:** Examples of pathology term qualifiers now incorporated into PATO

**Qualifier**	**PATO**	**Class name**	**Definition**
Severity	0000461	Normal	No lesions
	0000394	Mild	Lesion dependent; often size, number and characteristics.
	0000395	Moderate	
	0000465	Marked	
	0000396	Severe	
Duration	0002387	Per-acute	Extremely acute and aggressive
	0000389	Acute	Beginning abruptly with marked intensity
	0002091	Subacute	Between acute and chronic
	0001863	Chronic	Slow progress and long continuance
	0002387	Chronic-active	Coexistence of chronic process and superimposed acute process
Distribution	0000627	Focal	Single well delineated lesion
	0002388	Focally extensive	Single lesion with expansion into surrounding tissue
	0001791	Multifocal	Multiple lesions
	0002389	Multifocal to coalescing	Multiple lesions some interconnecting with each other
	0000330	Random	No appreciable pattern
	0001566	Diffuse	Not circumscribed or limited
	0000635	Generalized	Affecting all regions without specificity of distribution
	0000634	Unilateral	Confined to one side only
	0000618	Bilateral	Involving both sides
	0002389	Segmental	Relating to a segment

#### Implementation of MPATH coding strategy

The strategy adopted was originally designed to describe histopathology images for the Pathbase mouse pathology database [30], but lends itself readily to a wide range of coding applications. The MPATH strategy has been adopted by two major high-throughput studies. A combination of MPATH and PATO is being used for the capture of pathology data from the genome-wide mutant mouse phenotyping project, KOMP^2^ run as part of the International Mouse Phenotyping Consortium [31], where the MPATH approach is being used in the primary phenotyping pipeline by the Toronto Centre for Phenogenomics and other centres carrying out histopathology. MPATH has also been adopted for the MoDIS database [32] to capture and analyse pathology data from a massive aging study which has systematically phenotyped 31 of the most important inbred mouse strains. Complete necropsies of mice were carried out at 12 and 20 months of age (cross-sectional study) and moribund mice in the life span (longitudinal study). Nearly 2,000 mice were necropsied, generating more than 50,000 slides [33]. Lesion incidence and severity data for all organs is now being applied in highly successful GWAS studies of age-associated disease [33].

MPATH has proved to be additionally useful in dealing with the recoding of multi-species legacy data from non-standard nomenclatures, permitting integration of otherwise siloed data. Examples are the European Radiological archive (ERA) database where 6,700 human diagnoses were recoded from ICD-8 and the Klinischer Diagnosenschleussel [34] to MPATH/FMA [35], and with the Northwestern University Janus radiobiology database (http://janus.northwestern.edu/janus2/), who have coded 50,000 individual mouse records to MPATH to link the two datasets. Recently the ontology has been applied to zebrafish phenotype data in the Zfin database [36] indicating a useful application of MPATH to non-mammalian species which could be developed further.

#### MPATH as a core ontology for PATO-based logical definitions

The PATO framework was built with the intention of providing an integration platform for phenotype data between species and between data types [22]. According to the PATO framework, phenotype data can be described by utilising species-specific ontologies (such as the various anatomy ontologies) or species-agnostic ontologies such as GO with the various qualities provided by the PATO ontology in order to describe affected entities in a phenotype manifestation. PATO can be used for annotation either directly in a so-called post-composed (post-coordinated) manner or for providing logical definitions (equivalence axioms) to ontologies containing a set of precomposed (pre-coordinated) phenotype terms [22,37-39]. For further discussion see [40].

Rather than using a pre-composed phenotype ontology such as MP [29] or HPO [41], phenotypes may be described using the Entity–Quality (EQ) formalism. In the EQ method, a phenotype is characterized by an affected Entity and a Quality (from PATO) that specifies how the entity is affected. The affected entity can either be a biological function or process such as specified in GO, or an anatomical entity. The phylogenetic conservation, at least within the amniotes, of most histopathologic lesions or processes makes MPATH an important core ontology in writing logical definitions and we have used it extensively in defining classes in the major pre-composed phenotype ontologies and MPATH is an important component ontology of our recently developed semantic approaches to comparative phenomics – PhenomeNET and Mousefinder [8,9].

Composition of logical definitions is a time-consuming task for which there are currently several approaches to automation using class label segmentation, entity recognition and lexical matching to core ontologies. This approach can be useful for suggesting definitions where the class label is a composite of for example, anatomy and process (MA + GO). Automated decomposition of unilexical terms such as are found in the neoplasias is much more difficult though approaches with text mining definitions from other ontologies such as NCIt for lexically matching labels may be useful to expert curators in establishing more simple definitions for these classes.

## Conclusions

Whilst MPATH was originally designed to support rodent, and particularly mouse, pathology the extensive overlap with human pathology means that most of the terms may be used in a human context and linked to the foundational model of anatomy (FMA) [42] as the anatomy ontology. Extending MPATH to become a mammalian pathology ontology encompassing human pathology is a major undertaking, but we have established that the current structure and upper level classes would readily support the inclusion of human terminology. Initially we will import terms for neoplasias from the CINEAS codes (Central Information System for Hereditary Diseases and Synonyms; http://www.cineas.org/; Prof Rolf Sijmons, pers, comm). SNOMED-CT, UMLS and ICD-O v3 will be mined for terms not currently in MPATH which relate to anatomic pathology. Terms already covered by existing ontologies such as Disease Ontology (DO) [43] may be referenced using MIREOT [44]. DO classifies diseases largely by anatomical site and not by disease process or class, and overlaps only slightly with MPATH as it is concerned with summative diagnostic entities for the main part. For example there is no “inflammation” superclass in DO for the tissue specific inflammatory conditions described.

Use of MPATH to construct logical definitions for DO classes would potentially add a further dimension to the richness and applicability of DO.

The power of the description of pathological lesions to discriminate between diseases and therefore between models of human disease is substantial. We recently estimated the information content (IC) of pre-composed MP ontology terms used to code phenotypes in the EUMODIC mouse phenotyping pipeline [45], which included or excluded anatomic pathology descriptions, using their logical definitions. Pathology-related phenotypes were shown to have a significantly greater discriminatory power than other *in vivo* assays, strongly supporting the use of these assays in the development of mouse models of human diseases [13].

Further development and application of MPATH will inevitably depend on community engagement and we encourage anyone with an interest to provide feedback.

## Abbreviations

CINEAS: Central Information System for Hereditary Diseases and Synonyms; CL: Cell ontology; DO: Disease ontology; EMAP: EMouse atlas project; ERA: European radiobiology archive; EUMODIC: European mouse disease clinic; FMA: Foundational model of anatomy; GWAS: Genome-wide association study; HPO: Human phenotype ontology; ICD: International classification of disease; ICD-O: International classification of disease – oncology; IMPC: International Mouse Phenotyping Consortium; INHAND: International Harmonization of Nomenclature and Diagnostic Criteria for Lesions in Rats and Mice; KOMP2: Knockout mouse project 2; MA: Mouse anatomy ontology; MIREOT: Minimum information to reference an external ontology term; MMHCC: Mouse Models of Human Cancer consortium; MPATH: Mouse pathology ontology; MP: Mouse phenotype ontology; NCIt: National Cancer Institute Thesaurus; OBO: Open biological ontology; PATO: Phenotype and trait ontology; RITA: Registry of Industrial Toxicology Animal-data; SNOMED-CT: Systematized nomenclature of medicine clinical terms; STP: Society for toxicopathology; UMLS: Unified medical language system.

## Competing interests

The authors declare that they have no competing interests.

## Authors’ contributions

All of the authors have made major contributions to the writing and coding of the ontology and its definitions over many years. The paper was written by PNS, JPS, and GVG. All authors read and approved the final manuscript.

## References

[B1] Virchow R Cellular pathology as based upon physiological and pathological histology 1860 2 New York: R M DeWitt 10.1111/j.1753-4887.1989.tb02747.x 2649802

[B2] Plaut A Rudolf Virchow and today's physicians and scientists Bull Hist Med 1953 27 236 251 13051681

[B3] Hajdu SI Inventors of new laboratory and pathology terms Ann Clin Lab Sci 2007 37 192 194 17522378

[B4] Sundberg JP Schofield PN One medicine, one pathology, and the one health concept J Am Vet Med Assoc 2009 234 1530 1531 19527123 10.2460/javma.234.12.1530 PMC3804058

[B5] Taylor CR Hartsock RJ Classifications of lymphoma; reflections of time and technology Virchows Archiv 2011 458 637 648 21503765 10.1007/s00428-011-1083-0

[B6] Scheuermann RH Ceusters W Smith B Toward an ontological treatment of disease and diagnosis Summit Transl bioinformatics 2009 2009 116 120 PMC3041577 21347182

[B7] Washington NL Haendel MA Mungall CJ Ashburner M Westerfield M Lewis SE Linking human diseases to animal models using ontology-based phenotype annotation PLoS Biol 2009 7 e1000247 19956802 10.1371/journal.pbio.1000247 PMC2774506

[B8] Chen CK Mungall CJ Gkoutos GV Doelken SC Kohler S Ruef BJ Smith C Westerfield M Robinson PN Lewis SE MouseFinder: Candidate disease genes from mouse phenotype data Hum Mutat 2012 33 858 866 22331800 10.1002/humu.22051 PMC3327758

[B9] Hoehndorf R Schofield PN Gkoutos GV PhenomeNET: a whole-phenome approach to disease gene discovery Nucleic Acids Res 2011 39 e119 21737429 10.1093/nar/gkr538 PMC3185433

[B10] Oellrich A Hoehndorf R Gkoutos GV Rebholz-Schuhmann D Improving disease gene prioritization by comparing the semantic similarity of phenotypes in mice with those of human diseases PloS One 2012 7 e38937 22719993 10.1371/journal.pone.0038937 PMC3375301

[B11] Berndt A Cario CL Silva KA Kennedy VE Harrison DE Paigen B Sundberg JP Identification of *Fat4 * and *Tsc22d1* as novel candidate genes for spontaneous pulmonary adenomas Cancer Res 2011 71 5779 5791 21764761 10.1158/0008-5472.CAN-11-1418 PMC3165088

[B12] Li Q Berndt A Guo H Sundberg JP Uitto J A Novel Animal Model for Pseudoxanthoma Elasticum: The KK/HlJ Mouse Am J Pathol 2012 181 4 1190 1196 22846719 10.1016/j.ajpath.2012.06.014 PMC3463623

[B13] Schofield PN Vogel P Gkoutos GV Sundberg JP Exploring the elephant: histopathology in high-throughput phenotyping of mutant mice Dis Model Mech 2011 5 19 25 22028326 10.1242/dmm.008334 PMC3255539

[B14] Cornet R De Keizer N Forty years of SNOMED: a literature review BMC Med Inform Decis Mak 2008 8 Suppl 1 2 19007439 10.1186/1472-6947-8-S1-S2 PMC2582789

[B15] World Health Organisation International Statistical Classification of Diseases and Health Related Problems (The) ICD-10 2008 Geneva: WHO

[B16] Mann PC Vahle J Keenan CM Baker JF Bradley AE Goodman DG Harada T Herbert R Kaufmann W Kellner R International harmonization of toxicologic pathology nomenclature: an overview and review of basic principles Toxicol Pathol 2012 40 7S 13S 22637736 10.1177/0192623312438738

[B17] Kaufmann W Bolon B Bradley A Butt M Czasch S Garman RH George C Groters S Krinke G Little P Proliferative and nonproliferative lesions of the rat and mouse central and peripheral nervous systems Toxicol Pathol 2011 40 87S 157S 22637737 10.1177/0192623312439125

[B18] Frazier KS Seely JC Hard GC Betton G Burnett R Nakatsuji S Nishikawa A Durchfeld-Meyer B Bube A Proliferative and nonproliferative lesions of the rat and mouse urinary system Toxicol Pathol 2011 40 14S 86S 22637735 10.1177/0192623312438736

[B19] Thoolen B Maronpot RR Harada T Nyska A Rousseaux C Nolte T Malarkey DE Kaufmann W Kuttler K Deschl U Proliferative and nonproliferative lesions of the rat and mouse hepatobiliary system Toxicol Pathol 2010 38 5S 81S 21191096 10.1177/0192623310386499

[B20] Renne R Brix A Harkema J Herbert R Kittel B Lewis D March T Nagano K Pino M Rittinghausen S Proliferative and nonproliferative lesions of the rat and mouse respiratory tract Toxicol Pathol 2009 37 5S 73S 20032296 10.1177/0192623309353423

[B21] Marks C Mouse Models of Human Cancers Consortium (MMHCC) from the NCI Dis Model Mech 2009 2 111 19259381 10.1242/dmm.002725 PMC2650192

[B22] Gkoutos GV Green ECJ Mallon A-M Hancock JM Davidson D Building mouse phenotype ontologies Pac Symp Biocomput 2004 9 178 189 14992502 10.1142/9789812704856_0018

[B23] Gene Ontology Consortium The Gene Ontology in 2010: extensions and refinements Nucleic Acids Res 2010 38 331 335 10.1093/nar/gkp1018 PMC2808930 19920128

[B24] Bard J Rhee SY Ashburner M An ontology for cell types Genome Biol 2005 6 R21 15693950 10.1186/gb-2005-6-2-r21 PMC551541

[B25] Meuwissen R Berns A Mouse models for human lung cancer Genes Dev 2005 19 643 664 15769940 10.1101/gad.1284505

[B26] Derksen PW Liu X Saridin F van der Gulden H Zevenhoven J Evers B Van Beijnum JR Griffioen AW Vink J Krimpenfort P Somatic inactivation of E-cadherin and p53 in mice leads to metastatic lobular mammary carcinoma through induction of anoikis resistance and angiogenesis Cancer Cell 2006 10 437 449 17097565 10.1016/j.ccr.2006.09.013

[B27] Hayamizu TF Mangan M Corradi JP Kadin JA Ringwald M The adult mouse anatomical dictionary: a tool for annotating and integrating data Genome Biol 2005 6 R29 15774030 10.1186/gb-2005-6-3-r29 PMC1088948

[B28] Richardson L Venkataraman S Stevenson P Yang Y Burton N Rao J Fisher M Baldock RA Davidson DR Christiansen JH EMAGE mouse embryo spatial gene expression database: 2010 update Nucleic Acids Res 2010 38 D703 709 19767607 10.1093/nar/gkp763 PMC2808994

[B29] Meehan TF Masci AM Abdulla A Cowell LG Blake JA Mungall CJ Diehl AD Logical development of the cell ontology BMC Bioinform 2011 12 6 10.1186/1471-2105-12-6 PMC3024222 21208450

[B30] Schofield PN Gruenberger M Sundberg JP Pathbase and the MPATH Ontology: Community Resources for Mouse Histopathology Vet Pathol 2010 47 1016 1020 20587689 10.1177/0300985810374845 PMC3038412

[B31] Brown SD Moore MW Towards an encyclopaedia of mammalian gene function: the International Mouse Phenotyping Consortium Dis Model Mech 2012 5 289 292 22566555 10.1242/dmm.009878 PMC3339821

[B32] Sundberg JP Sundberg BA Schofield P Integrating mouse anatomy and pathology ontologies into a phenotyping database: tools for data capture and training Mamm Genome 2008 19 413 419 18797968 10.1007/s00335-008-9123-z PMC2844541

[B33] Sundberg J Berndt A Sundberg B Silva KA Kennedy V Bronson R Yuan R Paigen B Harrison D Schofield PN The mouse as a model for understanding chronic diseases of aging: the histopathologic basis of aging in inbred mice Pathobiol Aging Age-related Dis 2011 1 71719 10.3402/pba.v1i0.7179 PMC3417678 22953031

[B34] Immich H Klinischer Diagnosenschlüssel 1968 Stuttgart: Schattauer

[B35] Tapio S Schofield PN Adelmann C Atkinson MJ Bard JL Bijwaard H Birschwilks M Dubus P Fiette L Gerber G Progress in updating the European Radiobiology Archives Int J Radiat Biol 2008 84 930 936 19016141 10.1080/09553000802460214

[B36] Howe DG Bradford YM Conlin T Eagle AE Fashena D Frazer K Knight J Mani P Martin R Moxon SA ZFIN, the Zebrafish Model Organism Database: increased support for mutants and transgenics Nucleic Acids Res 2013 41 D854 860 23074187 10.1093/nar/gks938 PMC3531097

[B37] Gkoutos GV Mungall C Dolken S Ashburner M Lewis S Hancock J Schofield P Kohler S Robinson PN Entity/quality-based logical definitions for the human skeletal phenome using PATO Conf Proc IEEE Eng Med Biol Soc 2009 1 7069 7072 19964203 10.1109/IEMBS.2009.5333362 PMC3398700

[B38] Mungall CJ Gkoutos GV Smith CL Haendel MA Lewis SE Ashburner M Integrating phenotype ontologies across multiple species Genome Biol 2009 11 R2 20064205 10.1186/gb-2010-11-1-r2 PMC2847714

[B39] Gkoutos GV Mungall C Dolken S Ashburner M Lewis S Hancock J Schofield P Kohler S Robinson PN Entity/quality-based logical definitions for the human skeletal phenome using PATO Conf Proc IEEE Eng Med Biol Soc 2009 2009 7069 7072 19964203 10.1109/IEMBS.2009.5333362 PMC3398700

[B40] Schofield PN Sundberg JP Hoehndorf R Gkoutos GV New approaches to the representation and analysis of phenotype knowledge in human diseases and their animal models Brief Funct Genomics 2012 10 258 265 21987712 10.1093/bfgp/elr031 PMC3189694

[B41] Robinson PN Mundlos S The human phenotype ontology Clin Genet 2010 77 525 534 20412080 10.1111/j.1399-0004.2010.01436.x

[B42] Rosse C Mejino JL Jr A reference ontology for biomedical informatics: the Foundational Model of Anatomy J Biomed Inform 2003 36 478 500 14759820 10.1016/j.jbi.2003.11.007

[B43] Schriml LM Arze C Nadendla S Chang YW Mazaitis M Felix V Feng G Kibbe WA Disease Ontology: a backbone for disease semantic integration Nucleic Acids Res 2012 40 D940 946 22080554 10.1093/nar/gkr972 PMC3245088

[B44] Courtot M Frank G Allyson LL James M Daniel S Ryan RB Alan R MIREOT: The minimum information to reference an external ontology term Appl Ontol 2011 6 23 33

[B45] Morgan H Beck T Blake A Gates H Adams N Debouzy G Leblanc S Lengger C Maier H Melvin D EuroPhenome: a repository for high-throughput mouse phenotyping data Nucleic Acids Res 2012 38 D577 585 19933761 10.1093/nar/gkp1007 PMC2808931

